# Influence of angles occlusion in periodontal diseases

**DOI:** 10.6026/97320630016983

**Published:** 2020-12-31

**Authors:** Sheeja S Varghese

**Affiliations:** 1 Department of Periodontics, Saveetha Dental College and Hospitals, Saveetha Institute of Medical and Technical Sciences, Saveetha University 162, Poonamallee High Road, Chennai-600077, India

**Keywords:** Age, angles malocclusion, gender, gingivitis, periodontitis

## Abstract

It is of interest to document the known relationship between periodontal status and Angle's malocclusion types. We used 26092 case records of patients between 16 to 50 years of age with no gender restrictions. Variables such as age, gender, periodontal diagnosis and type of Angle's occlusion were extracted and tabulated. Statistical analysis was completed using chi square test in the SPSS software version 20. Data shows that the majority (95.27%) had Angle's class I occlusion and less than 5% had class II and Class III occlusion. Statistical analysis of class II and Class III cases with 1000 randomly selected cases of class I occlusion show a significant difference in the periodontal status between different types of Angle's occlusion. Chronic periodontitis was more in class I (10.4%) and it was the lowest in Class II Div 2 (4.3%) occlusion. Class II Div 1(23.8%) and Class III (17%) had the highest and lowest proportion of clinically healthy periodontium, respectively. Thus, we report that angles occlusion types had significant influence on periodontal status along with the other determinants.

## Background:

Gingivitis and periodontitis are the two common chronic inflammatory diseases affecting the supporting structures of the teeth and the former leads to the latter and eventually causes tooth loss [1,2]. Even though microorganisms associated with dental plaque are considered as the major etiological factor [3,^4^] other local and systemic contributing factors also play a role in the initiation and progression of these diseases [5-8]. Dental occlusion is implicated as a contributing factor in the pathogenesis of periodontitis [9]. The causal relationship between dental occlusion and periodontitis is still inconclusive due to contradictory reports [10,11]. Angle's classification, developed a century ago, is one of the major classifications used to categorize the malocclusions and it mainly uses the first molar relationship of maxillary and mandibular arch along with canines and maxillary anteriors [12]. The malocclusion is implicated in the etio-pathogenesis of periodontal disease primarily due to its influence on oral hygiene maintenance leading to more plaque accumulation but its influence on trauma from occlusion is noteworthy [13,14,10]. A lot of research has been carried out in the past and has reported the influence of malocclusion on periodontal health and diseases. Conversely, periodontal health can influence the occlusion [15]. Pathological tooth migration resulting from periodontitis can cause occlusal disharmony [16]. Therefore, it is of interest to document the known relationship between periodontal status and Angle's malocclusion types.

## Material and Methods:

This retrospective study included patients undergoing treatment in a dental hospital from June 2019 to March 2020. The scientific review board approved the study and ethical clearance was obtained from the Institutional Ethical Committee of the university (SDC/SIHEC/2020/DIASDATA/0619-0320). Over 86000 case records were downloaded from the Digital Archives System of the hospital. Consecutive sampling was used for including the cases using the following inclusion and exclusion criteria. Patients between the age group of 16 - 50 with complete periodontal examination and orthodontic examination records were included, irrespective of gender. Periodontal examination should be with 6 site probing along with gingival inflammation examination and periodontal diagnosis based on AAP 1999 criteria. Orthodontic examination should have molar occlusion details with Angle's classification of malocclusion. Patients with incomplete records, case records which were not approved by the respective specialist, cases with severe deformities such as cleft palates, case records where Angle's classification was not recorded due to many missing teeth, cases with traumatic injuries and cases with oral cancer or any other systemic diseases were excluded. Variables collected included the age of the patient, gender, type of occlusion based on Angle's classification, periodontal diagnosis. The periodontal diagnosis types included clinically healthy, chronic gingivitis (localised or generalised) and periodontitis (localised or generalised). The data was tabulated and descriptive analysis was done using percentages and statistical analysis was done using SPSS software version 20. Chi square test was used to analyse the association between occlusion and periodontal status and the association of gender and age on periodontal and occlusal parameters. P value less than 0.05 was considered as statistically significant.

## Results:

The study included a total of 26092 patients out of which 15044(57.66%) were males 11044 (42.33) were females and 4(0.02%) were transgenders. Majority (95.27%) of them had Angle's class I occlusion (24858), 853 (3.27%) of them had class II Div 1 occlusion, 117 (0.45%) had class II Div 2 occlusion and 264(1.01%) had class III occlusion (Figure 1 - see PDF). Since there was a huge variation in the sample size between class 1 occlusion and other types of occlusion, for statistical analysis, a sample of 1000 patients from class I occlusion was randomly selected along with all the patients belonging to class II and class III occlusion. Thus a total of 2234 patients were included for the statistical comparison (Figure 1 - see PDF). In this selected sample 44.8% were with class I occlusion, 38.2% with class II Div 1 occlusion, 5.2% with class II Div 2, and 11.8% were with class III occlusion. On evaluating the periodontal status, 21.2% were clinically healthy, 9% were with localised chronic gingivitis, 58.9% were with generalised chronic gingivitis, 3.2% were with localised chronic periodontitis and 7.7% with generalised chronic periodontitis (Table 1 and Table 3 - see PDF). The selected sample had 45.3% females and 54.7% males. On analysing whether molar occlusion had any influence on periodontal status, the results showed that in all the four types of occlusions, patients with generalised chronic gingivitis were more in number followed by patients with clinically healthy gingiva. The other types of periodontal disease categories were comparatively less in number. While analysing the periodontal status between the different types of Angles occlusion, it was observed that clinically healthy gingiva was comparatively more in class II Div 1 occlusion as compared to other types of occlusion and class III had the lowest number. Further periodontitis was comparatively more in class I occasion as compared to other types of occlusion and class II Div 2 had the lowest number. Statistical analysis was performed using chi square test and it was found that the difference was significant with a p value of 0.001 (Table 1 and Figure 2 - see PDF). On analysing the gender wise comparison of periodontal status, the observations were as follows. Comparatively more females had clinically healthy gingiva than males and periodontitis was comparatively more in males. But the difference was not statistically significant (p value 0.06) (Table 2 and Figure 3 - see PDF). Gender wise comparison of occlusion revealed that more females had class II Div 1 occlusion than males whereas more males had class III occlusion than females. This difference was also statistically significant with p value less than 0.001. (Table 3 and Figure 4 - see PDF). While analysing the mean age of the patients with respect to periodontal status, the observations were as follows. The mean age of the group with clinically healthy gingiva was 26.40±8.35 and the gingivitis group, both localised and generalised, had the mean age 29.42±8.4 and 28.96±8.65. Localised chronic periodontitis had the mean age of 35.2±8.98 and the highest mean age was for the generalised chronic periodontitis group with the mean age of 40.86±7.28. This difference in the mean ages was also statistically significant p value <0.001 (Table 4 and Figure 5 - see PDF).

## Discussion:

The results of the study revealed that more males had visited the hospital than females for dental treatment and transgenders seeking dental treatment was very rare. On the contrary, it has been reported from a study done in Dental training school in Wisconsin that females were more predominant than males in seeking preventive dental treatment [17] The present study included both those who came for preventive as well as corrective treatments. Another observation was that only very few transgenders (0.02%) had reported for dental treatment. Even though the transgender population in Chennai is around 0.3%, the percentage distribution in patients reporting for dental treatment was very low. Health care utilisation was comparatively less in transgenders as compared to cisgenders [18]. More awareness programs are needed to break the barriers in this aspect. While analysing the prevalence of different types of Angle's occlusion from the entire data, the observations were that the majority of the patients had class I occlusion and less than 5% had class II and class III occlusion put together. Among class II and III occlusion, the predominant type was class II Div 1, followed by class III and the least common was class II Div 2. On the contrary, a study done in an orthodontic clinic reported that nearly 50% of the patients who visited had class II malocclusion, 33% had class I malocclusion and 32% had class III malocclusion [19]. Variability between the two results could be due to the fact that the present study included patients from a general dental hospital. In the present study, class III occlusion was the least predominant type. Supporting this finding, a systematic review and Meta analysis on prevalence of class III malocclusion reported that Indians had a lower prevalence of class III malocclusion than other racial groups [20].

The analysis of the periodontal status revealed that nearly 68% of the included sample had gingivitis in either the localised or generalised form, 21% had clinically healthy gingiva and only around 11% had periodontitis. Gingival disease experience in Australian adult population was reported to be only 19.7% [21] and this is very low as compared to the gingivitis prevalence observed in the present study. But a major difference with respect to our study was that the previous study did not include patients having gingival index scores less than 2. This might be the reason for the lower prevalence rate as compared to the present study results. In the same Australian adult population, the Periodontitis prevalence was 22.9%, which is higher than the prevalence rate observed in the present study. Similar to our study, the same AAP criteria were used for the periodontitis diagnosis in the study [21]. In the Indian population, various studies have reported a prevalence rate ranging from 41% to 75% for periodontitis and it is very much higher than the present study results. The two major reasons for lower prevalence are that in the present study, the inclusion criteria restricted patients from 16 to 50 yrs of age. Most of the previous studies had included adult patients even up to 75 years [22]. Since the main aim of the present study was to explore the orthodontic and periodontal relationship, the study also excluded patients whose Angles occlusion is not recorded due to many missing teeth. This may have also eliminated some of the severe periodontitis cases.

On analysing whether there is an association between occlusion and periodontal status, the observations were that clinically healthy periodontium was proportionately more in class II Div 1 occlusion and least in class III occlusion. With respect to periodontitis, class I had proportionately more numbers and class II Div 2 had the lowest numbers. The association between the type of occlusion and periodontal status was statistically significant. There was no previous study, which compared the periodontal status between different Angle's malocclusion to compare with the results of the present study. It has been reported that the amount of alveolar bone loss in mandibular anterior teeth was significantly more in class III occlusion as compared to class I occlusion [23] when evaluated with CBCT. Another interesting report was that there was a significant difference in the inter maxillary tooth size discrepancy between Class I, Class II and Class III type of occlusion with the ratio being highest for class III followed by Class I and lowest in ClassII [24]. A study which compared the Plaque index and Gingival index between different types of Angles malocclusion reported statistically significant but mild variation between Class I, Class II and Class III occlusion with Class I having comparatively more proportion of people with highest score of 3 in both the indices [25]. Moreover, a population based cross sectional study also concluded that malocclusions and morphologic parameters were associated with periodontal diseases [25-26]. The association of periodontal status and Angles occlusion observed in the study needs further research to elucidate the causal relationship between them.

Males were reported to have more periodontal destruction than females in many studies [27,28]. The results of the present study showed that males had more generalised chronic periodontitis than females and clinically healthy gingiva was more prevalent in females than males. But in statistical analysis the difference was not significant. This study also observed that class II Div 1 occlusion was more prevalent in females whereas class III was more in males. Supporting this finding, a study reported that Angle's class III was more prevalent in females and Angles Class III and Class II were more prevalent in males [29]. It has also been reported that sexual dimorphisms are more in class III malocclusion [29,30]. Gender differences had been reported in Indian population as well [31]. The results of the study clearly indicate the relationship of age and periodontal diseases. The mean age was lowest in the clinically healthy group and it was gradually increased to the highest mean value for the generalised chronic periodontitis group. This is in accordance with the previous reports that say that age is a major determinant in periodontitis and as the age increases the risk of periodontitis increases [32]. A recent ecological study on global prevalence of periodontal diseases, which included data from the World Health Organisation data bank, also concluded that the distribution of periodontal disease increases with age [33]. This study analysed the relationship between the Angles occlusion and periodontal status along with the age and the gender determinants. The respective specialist with established protocols did periodontal and orthodontic diagnoses. The sampling technique used was consecutive sampling which eliminated the selection bias. But being a retrospective study it has inherent limitations. Moreover, other risk factors for periodontitis such as oral hygiene, smoking and systemic status were not considered. The larger sample size could reduce these biases to a certain extent. The results of the study reveal an association between periodontal status and Angle's molar occlusion, which needs further longitudinal research. The results also prove the influence of gender and age on periodontitis and the influence of gender on malocclusion.

## Conclusion:

We report that Angles occlusion types had significant influence on periodontal status along with the other determinants. The results of the study are adding more insight into the orthodontic periodontal interrelationships. 

## Author contribution:

S.V contributed to study conception and design, data collection, analysis, interpretation and manuscript preparation.
